# Digital self-management for chronic disease care: a mini review of service integration, equity, and public health implementation priorities

**DOI:** 10.3389/fpubh.2026.1883085

**Published:** 2026-10-16

**Authors:** Fanchang Wang, Hongyang Qiao, Yuqin Wu, Xiaoming He, Lan Ying

**Affiliations:** 1 The Second School of Clinical Medicine, Zhejiang Chinese Medical University, Hangzhou, China; 2 The First Affiliated Hospital of Wenzhou Medical University, Wenzhou, China; 3 The Second Affiliated Hospital of Zhejiang Chinese Medical University, Hangzhou, China

**Keywords:** chronic disease, digital health, health equity, implementation, integrated care, large language models, remote patient monitoring, self-management

## Abstract

Digital self-management is increasingly used to support people with chronic non-communicable diseases between visits, but its public health value depends on integration into routine care. This SANRA-informed critical narrative mini review synthesized 28 sources identified through PubMed/MEDLINE, backward citation tracking and targeted supplementary searches. It examines digital self-management as service redesign across three delivery models: patient centered digital support, remote patient monitoring, and integrated primary care or community coordination. It also analyzes three implementation tensions: empowerment versus digital exclusion, data visibility versus clinical accountability, and engagement versus uncertain long term outcomes and workload. Evidence suggests benefits for engagement, behavioral support and selected intermediate indicators, but these benefits remain uneven. Public health implementation should therefore prioritize accountable workflows, interoperable procurement, equity oriented support, safe use of large language model based agents, and evaluation of outcomes, safety, equity, cost and workforce burden. Digital self-management should be judged by whether it supports routine, inclusive and sustainable chronic disease care.

## 1 Introduction

Chronic non-communicable diseases (NCDs), especially cardiovascular disease, cancer, chronic respiratory disease and diabetes, remain leading causes of death, disability and health system pressure worldwide. Global Burden of Disease 2021 projections indicate that deaths and disability adjusted life years attributable to NCDs will continue rising toward 2050, with cardiovascular disease making a major contribution (1). The economic burden is also substantial, with NCDs estimated to cost the global economy USD 47 trillion between 2011 and 2030 (2).

Effective chronic disease management relies heavily on what patients do between clinical visits—taking medications, monitoring symptoms, measuring physiological indicators, adjusting lifestyle behaviors and seeking help when needed. Self-management is therefore a core part of chronic care, although in many health systems it is still weakly supported, unevenly reimbursed and poorly linked to routine clinical workflows. Digital tools—including mobile applications, web portals, wearables, conversational agents and remote patient monitoring devices—may help extend support beyond the clinic (3), capture patient generated data and provide more tailored education or behavioral prompts.

The field has developed rapidly, but evidence for population level impact remains mixed (4). Interventions may improve engagement, confidence and selected intermediate indicators, whereas effects on long term outcomes, equity, clinician workload and sustainability remain less clear. Previous reviews have described digital health platforms, assessed European primary care effectiveness and mapped implementation patterns (5–7). This mini review instead examines digital self-management as service redesign, asking which delivery models, accountability arrangements and implementation conditions are needed for routine, equitable and sustainable chronic care.

## 2 Scope and organizing framework

This article is a critical narrative mini review, prepared in line with SANRA guidance, and should not be read as a systematic review or meta-analysis. An iterative PubMed/MEDLINE search was conducted between January and March 2026. Eligible sources included English-language peer-reviewed publications and public health or policy-oriented documents, mainly from January 2020 to March 2026, with seminal earlier sources retained when relevant. Search concepts combined digital self-management or digital health with chronic disease or non-communicable disease and implementation-related terms. Priority was given to reviews, implementation studies, real-world evaluations, management-relevant trials and policy analyses. Protocols without findings, commentaries, conference abstracts and purely technical studies without workflow or equity relevance were excluded. The synthesis was thematic, with limitations discussed in Section 6.

The search retrieved 60 records. After title and abstract screening, 25 were excluded and 35 underwent detailed assessment. Twelve were subsequently excluded—protocols without findings or commentaries (*n* = 5), and technical or algorithmic studies without workflow or equity relevance (*n* = 7)—leaving 23 PubMed/MEDLINE sources. Backward citation tracking added two sources and targeted supplementary searches added three, yielding 28 sources: 27 journal publications and one institutional report. Supplementary Figure S1 summarizes the process. Because this was a narrative rather than a systematic review, the figure is descriptive rather than a formal PRISMA-compliant flow diagram.

The organizing framework for this review is presented in Figure 1. It links digital self-management tools with three service delivery models: patient-centered digital support, remote patient monitoring, and integrated primary care or community coordination. These categories were developed through thematic synthesis of prior reviews, implementation studies and integrated care frameworks, and are used as an organizing structure rather than a validated taxonomy. Across these models, public health value depends on six implementation conditions: equity and access, support for health and digital literacy, interoperability, privacy and governance, sustained engagement, and alignment of workforce and financing. The central argument is that digital tools do not, by themselves, generate population health gains. They require accountable care models and practical implementation conditions before they can become chronic care infrastructure.

**Figure 1:**
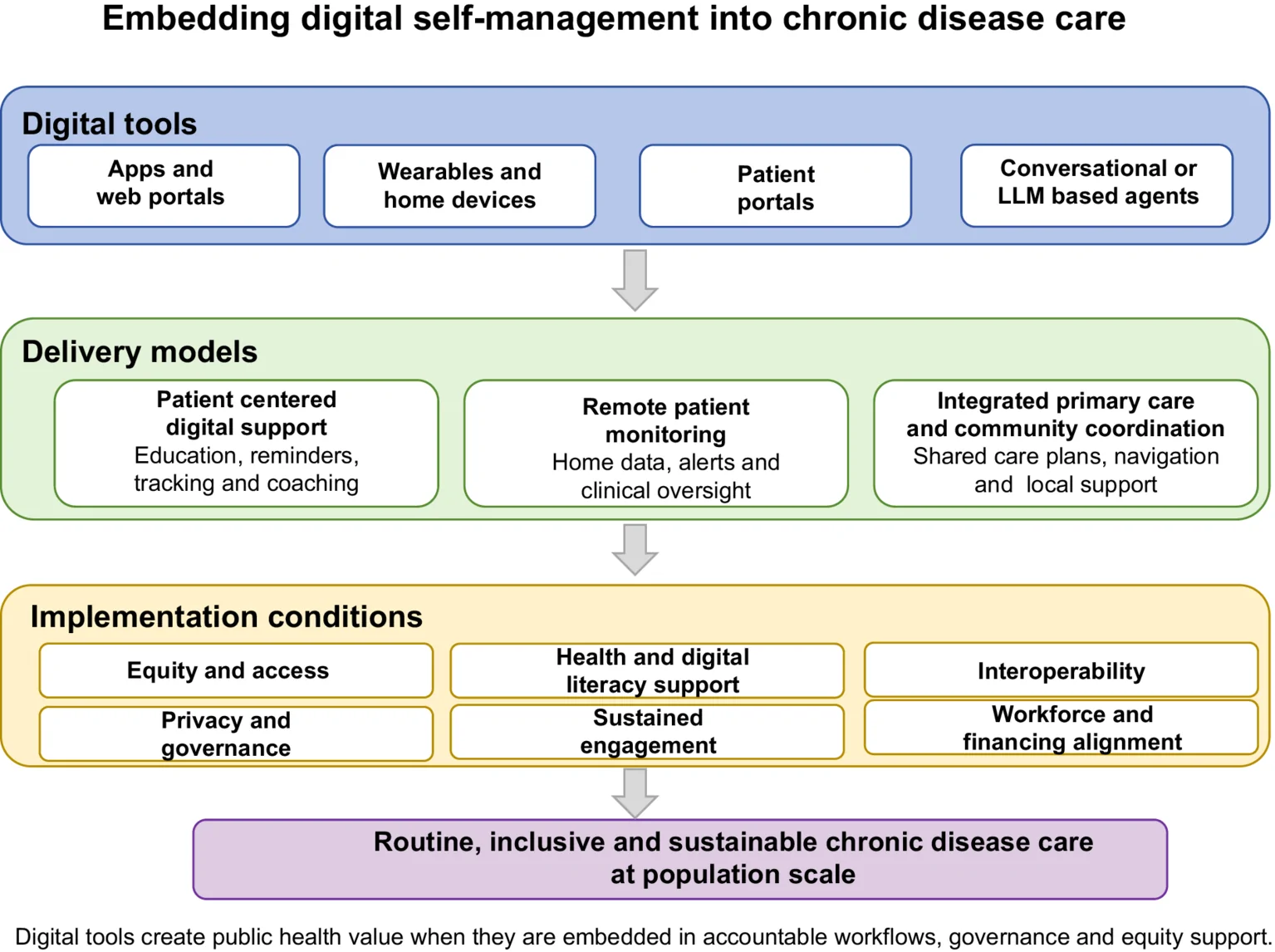
Management framework for embedding digital self-management into chronic disease care. The framework shows how apps, web portals, wearables, conversational or large language model-based agents and remote patient monitoring devices can be organized through patient-centered support, remote patient monitoring, and integrated primary care or community coordination. It also shows that equity and access, health and digital literacy, interoperability, privacy and governance, sustained engagement, and alignment of workforce and financing are required to translate digital tools into routine, inclusive and sustainable chronic care at population scale.

Equity and access refer to whether digital self-management can be reached and used by groups with different socioeconomic, geographic, linguistic and disability-related needs. Health and digital literacy refer to patients’ ability to understand digital feedback and act safely. Interoperability concerns data exchange with clinical records and workflows. Privacy and governance cover data protection, vendor accountability, clinical oversight and responsible AI use. Sustained engagement refers to continued use beyond onboarding. Workforce and financing alignment refers to staffing, reimbursement and role allocation that make digital self-management feasible in routine care rather than only in short-term projects.

## 3 Management models for digital self-management

Digital self-management should not be understood as a single intervention. It is delivered through overlapping models that differ in responsibility, patient-generated data flows and links to professional care. Three models are especially relevant for public health systems.

Table 1 maps each delivery model against the six implementation conditions. The subsections then summarize evidence and management implications for each model.

**Table 1:** Relationship between digital self-management delivery models and implementation conditions.

Delivery model	Equity and access	Health and digital literacy	Interoperability	Privacy and governance	Sustained engagement	Workforce and financing
Patient-centered digital support	Risk of exclusion if apps require devices, broadband, language proficiency or high confidence with technology.	Requires plain-language education, coaching and digital navigation.	Often weakly linked to EHRs and routine clinical decision-making.	Requires clear clinical scope, accountability and safeguards, especially for LLM-based agents.	App attrition and declining use are common after initial onboarding.	Low marginal cost, but reimbursement and staff responsibility are often unclear.
Remote patient monitoring	May benefit high-risk patients, but can exclude those lacking devices, connectivity or stable home environments.	Requires training on device use, measurement technique and response to alerts.	Needs dashboards, EHR integration and actionable data summaries.	Requires response rules, data governance, vendor oversight and escalation protocols.	Engagement depends on feedback loops and perceived usefulness.	Adds alert review, message triage, documentation and follow-up workload.
Integrated primary care/community coordination	Strongest potential for equity when supported by primary care, community workers and local outreach.	Can provide culturally adapted education and human support.	Requires shared care plans and information exchange across organizations.	Needs role clarity across teams and organizations.	May support long-term adherence through local relationships.	Requires sustainable team-based payment and protected staff time.

### 3.1 Patient centered digital support

This subsection synthesizes sources (8–14), including platform reviews, LLM reviews and AI governance analyses.

The most common model relies on mobile applications and web platforms to help patients monitor symptoms, medications, physical activity, diet, mood and treatment goals. One systematic scoping review identified 69 digital health platforms for self-management across approximately 20 chronic conditions, most of which were mobile applications or combined web and mobile tools (8). From a management perspective, patient centered tools are attractive because they can be scaled relatively easily and at low marginal cost. Their main weakness, however, is that they may function alongside routine care rather than within it, providing information and motivation without a clear pathway into clinical decision making.

Conversational agents expand this model by offering interactive education, reminders and coaching (9). Earlier systems were often rule based and unevenly evaluated. Since 2024, large language model based agents have enabled more flexible explanations, medication questions, visit preparation and behavioral support (10). They also introduce safety concerns, including hallucinated advice, unsafe self-triage, bias, privacy leakage and unclear accountability (11). These concerns intersect with evolving frameworks for software as a medical device and AI enabled health products (12, 13). Chronic disease applications therefore need explicit clinical scope, escalation rules, human oversight and post-deployment monitoring (14).

### 3.2 Remote patient monitoring

This subsection synthesizes sources (15–18), including RPM reviews, implementation reports and outcome studies.

Remote patient monitoring (RPM) connects self-management with clinical oversight by sending blood pressure, glucose, weight, oxygen saturation, symptoms or other patient reported data from the home to care teams (15). Programmes such as the Mayo Clinic nurse based RPM model illustrate how devices can be paired with structured nursing oversight and integration into electronic health records (16). Some RPM studies have reported improvements in selected utilization outcomes—including reductions in hospitalizations or emergency department visits in groups such as patients with chronic obstructive pulmonary disease (17) or older adults with polypathology (18)—but effects vary by population, workflow design, monitoring intensity and outcome definition.

The main debate is whether RPM primarily prevents avoidable deterioration or moves clinical work into less visible digital channels. Alerts, asynchronous messages and trend reviews can add considerable workload when thresholds, escalation rules and documentation responsibilities are not clearly defined. RPM is therefore most useful when patient generated data are linked to agreed clinical actions, rather than collected continuously for monitoring alone.

### 3.3 Integrated primary care and community coordination

This subsection synthesizes sources (19–22), including integrated-care, RPM and primary-care digital transformation literature.

A third model places digital self-management within integrated care and local coordination. The SELFIE framework for integrated care for multimorbidity emphasizes service delivery, leadership, workforce, financing, technologies, information and research across micro, meso and macro levels (19). A scoping review of RPM from an integrated care perspective highlighted the importance of multidisciplinary teams, shared care plans, agreed response protocols and financing arrangements (20). Nurse led digital support may be effective, but only when roles, time allocation and escalation pathways are clearly defined.

Primary care and community based coordination are particularly important from a population health perspective (21). Recent analyses of digital transformation in primary health care, including the integration of large language models, have identified interoperability, AI enabled decision support and workforce training as key enablers, while infrastructure gaps and limited digital health literacy remain important barriers (22). Community health workers, health coaches and digital navigators can help bring digital self-management to patients who might otherwise be left out. Although this model is less technologically prominent than AI or RPM, it may be one of the most important approaches for advancing equity.

## 4 Controversies and implementation gaps

The main disagreements in the field are not about whether digital tools can help some patients. They concern which patients benefit, which services become responsible, and which outcomes should define success.

The implementation gaps below were identified by comparing the three delivery models with the six implementation conditions. Priority was given to recurring gaps with direct implications for equity, patient safety, clinical accountability, sustainability or public health scale-up. This approach emphasizes gaps that affect implementation across models, rather than isolated problems reported in single technologies or studies.

### 4.1 Empowerment versus exclusion

This theme draws mainly on sources (23, 24) on digital health equity and strategies for inclusive implementation.

One view is that digital self-management can empower patients by improving access to information, feedback and support. A more cautious view is that rapid digitalization may widen inequalities when programmes assume that patients have access to devices, broadband connectivity, English language proficiency, confidence with apps and stable living conditions. Uptake of digital health tools is often higher among urban, economically advantaged and digitally confident groups, whereas older adults, rural communities, minority groups and low income patients are less consistently represented in the evidence base (23).

This gap cannot be addressed through better interface design alone. Effective use also depends on health literacy, digital literacy and trust. Patients need to understand feedback, respond appropriately to alerts and know when to seek help. Implementation studies suggest that coaching, navigation, culturally tailored education and trusted local support are important for equitable digital health adoption (24). A remaining research question is how these human supports can be scaled without making digital programmes too costly or dependent on short term project funding.

Mitigating socioeconomic digital exclusion requires more than simpler interfaces. Programmes should consider device lending or subsidies, low-bandwidth and mobile-first designs, multilingual plain-language materials, telephone or in-person alternatives, and digital navigator support through primary care or community organizations. Without such supports, digital self-management may preferentially serve patients who already have greater resources.

### 4.2 Data visibility versus clinical accountability

This theme draws on sources (12–14, 20, 25, 26) on interoperability, governance and AI safety.

Digital self-management generates more data, but more data do not necessarily make care safer. Without usable exchange with electronic health records, platforms may expand monitoring without giving clinicians actionable summaries. Interoperability standards, open application programming interfaces and shared data models should therefore be treated as management requirements rather than technical preferences (20, 26).

Privacy and governance are equally important. Home, app and sensor data raise questions about access, response expectations, vendor use and responsibility for unsafe automated advice. These concerns are sharper for AI-enabled and large language model-based tools, where model updates, hallucinated advice, bias and unclear escalation may affect safety and trust (12–14, 25). Governance should specify data access, auditability, clinical scope, escalation thresholds, human oversight and post-deployment monitoring. Without these arrangements, data visibility may increase institutional risk without improving patient safety.

### 4.3 Engagement and intermediate benefit versus durable outcomes and workload

This theme draws on sources (4, 6, 15, 20, 27) on engagement, effectiveness and workload.

Sustained engagement remains a major weakness of digital self-management interventions (4, 6, 27). Use may decline within months, even when reminders, gamification and tailored feedback are included. This matters because chronic disease care depends on stable routines rather than short periods of initial enthusiasm.

Workload is not limited to reviewing abnormal values. Digital self-management can create work through onboarding, troubleshooting, message triage, alert review, documentation, follow-up calls, medication adjustment and responsibility for visible but not immediately actionable data. If these tasks are not assigned to specific staff and supported by reimbursement, monitoring may shift work into asynchronous channels without reducing face-to-face demand. Workload should therefore be evaluated through message volume, alert burden, response time, staff time, escalation frequency and clinician experience.

The evidence on effectiveness is also uneven. A randomized trial of an internet-based self-management and symptom monitoring programme for multiple chronic diseases did not significantly reduce all-cause hospitalizations over 2 years, although some secondary outcomes were favorable (27). A meta-analysis of European primary care trials similarly found no significant differences from usual care for most outcomes, except a small improvement in systolic blood pressure (6). These findings do not argue against digital self-management. They show that implementation should be assessed by clinical value, workload, safety and equity, not adoption metrics alone.

## 5 Public health implementation priorities

For managers, commissioners and policymakers, the practical question is not whether to buy digital tools, but how to embed them safely and fairly into chronic care.

The priorities below follow from the preceding analysis. Workflow redesign addresses accountability and workload problems. Procurement for integration, safety and governance addresses interoperability, privacy and AI accountability gaps. Evaluation of value, equity and sustainability addresses digital exclusion, declining engagement, uncertain long-term outcomes and workforce burden.

### 5.1 Redesign workflows around accountable action

Programmes should define the pathway from patient-generated data to clinical action, including eligibility, onboarding, monitoring frequency, alert thresholds, escalation routes, documentation responsibilities and stopping rules. Staff responsibilities should be specified for nurses, pharmacists, community health workers, digital navigators and physicians. Workload measures, including message volume, alert review time and response time, should be tracked alongside patient outcomes.

For illustration, a hypertension RPM programme might include adults with uncontrolled blood pressure taking two or more antihypertensive agents. A nurse or pharmacist could lead onboarding, device training and digital literacy support (28). Weekly readings could feed a shared dashboard, with pharmacist-led medication titration under an agreed protocol, physician escalation for predefined safety thresholds, multidisciplinary review at 3 months and de-escalation once control is sustained. The device is visible, but the workflow creates value. It determines whether the programme can be safe, equitable and sustainable at scale.

### 5.2 Procure for integration, safety and governance

Procurement should prioritize tools that exchange data with existing records, support open standards and provide transparent rules for data storage, vendor access, model updates and auditing. For AI and large language model-based agents, contracts should specify clinical scope, evidence sources, escalation behavior, privacy protections, bias monitoring and post-deployment surveillance.

### 5.3 Evaluate value, equity and sustainability

Evaluation should include implementation, clinical and patient-reported outcomes, safety signals, equity indicators, cost and workforce burden. RE-AIM and the Consolidated Framework for Implementation Research can help distinguish intervention failure from implementation failure. Outcomes should be stratified by age, language, socioeconomic status, rurality, disability and other local equity indicators. Programmes should also be allowed to fail in evaluation when they do not deliver value. This is necessary if digital self-management is to mature from promising pilots into public health infrastructure.

## 6 Discussion

This mini review shows that digital self-management for chronic disease care should be understood as service redesign, not as stand-alone technology. Patient-centered support may extend education, reminders and coaching, but value is limited when access, literacy and accountability are weak. RPM can improve data visibility and enable earlier response, but it creates responsibilities for alert review, escalation and documentation. Integrated primary care and community coordination may be less technologically visible, but they remain central to equity, literacy support and sustainable chronic care. Across models, the six implementation conditions determine whether digital self-management becomes routine, inclusive and sustainable.

This mini review has limitations. It did not involve a fully systematic search, formal risk-of-bias assessment or quantitative synthesis. The evidence base consists mainly of English-language sources and is drawn largely from high-income health systems. Published interventions may also differ from routine practice, where infrastructure, staffing, reimbursement and digital literacy vary considerably. Because the field is changing quickly, particularly with the emergence of large language model-based agents, conclusions about specific technologies will need updating as new evidence becomes available.

Future work should prioritize four areas. First, generative AI and large language model-based agents need evaluation for safety, hallucination, bias, escalation behavior, clinician workload and patient understanding (25). Second, privacy-preserving and interoperable infrastructure should be assessed for governance feasibility, not only technical performance (26). Third, reimbursement should support team-based review, patient education, digital navigation and proactive outreach. Fourth, pragmatic evaluations should assess clinical outcomes, patient-reported outcomes, cost, workload, alert fatigue and inequity.

Digital self-management has become a credible part of chronic disease care, but its public health value remains conditional. It will contribute most when health systems treat it as service redesign rather than technology procurement. Its contribution will be weakest when adoption moves faster than the workflows, financing, governance and equity supports needed for chronic care infrastructure. The key question is no longer only whether these tools work under trial conditions, but whether systems can build the workflows, governance and equity supports needed for high-burden populations. The argument advanced here is therefore not against digital tools, but for service integration and accountability.
