# Supplementary material for: Digital self-management for chronic disease care: a mini review of service integration, equity, and public health implementation priorities
Source: Front Public Health. 2026 Oct 16;14:1883085. doi: 10.3389/fpubh.2026.1883085 (PMC13720902; doi:10.3389/fpubh.2026.1883085)
Supplement: Supplementary file 1 [file Data_Sheet_1.pdf]

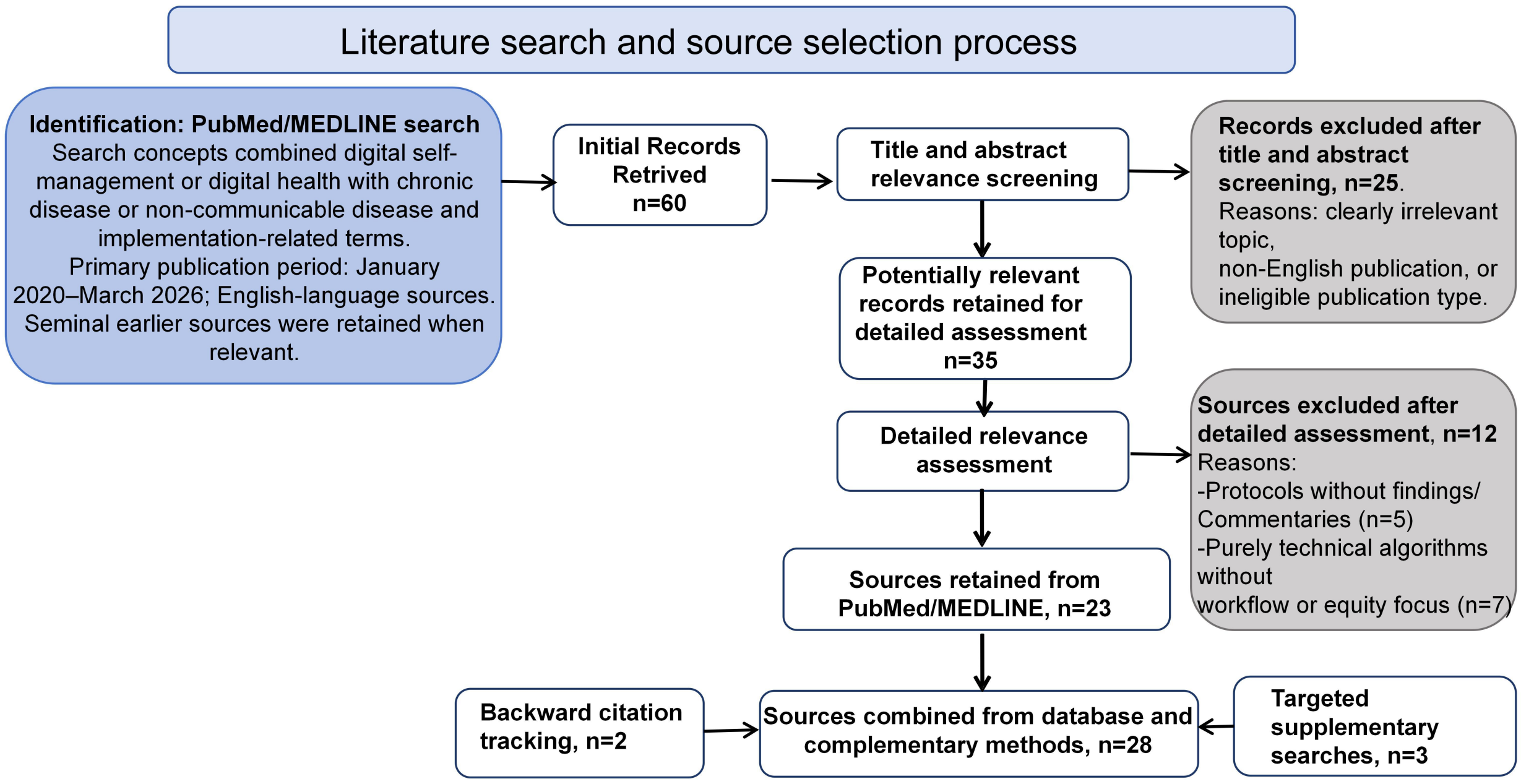

Figure S1. Literature search and source selection process. An iterative PubMed/MEDLINE search identified 60 records. After title and abstract relevance screening, 35 records underwent detailed assessment and 23 sources were retained from PubMed/MEDLINE. Backward citation tracking identified two additional sources and targeted supplementary searches identified three, resulting in 28 sources included in the narrative synthesis. The diagram is descriptive and does not represent a formal PRISMA-compliant systematic review flow.
